# Evaluation of the efficacy and safety of lixisenatide add-on treatment to basal insulin therapy among T2DM patients with different body mass indices from GetGoal trials

**DOI:** 10.1186/s13098-015-0104-6

**Published:** 2015-11-21

**Authors:** Kazuhiro Eto, Yusuke Naito, Yutaka Seino

**Affiliations:** Department of Internal Medicine, School of Medicine, Teikyo University, Tokyo, Japan; Sanofi K.K., Tokyo, Japan; Kansai Electric Power Hospital, Osaka, Japan

**Keywords:** Body mass index, Lixisenatide, Type 2 diabetes mellitus

## Abstract

**Background:**

Using patient data from the GetGoal-Duo1, -L, and L-Asia trials, the objectives of this study were to evaluate and compare the impact of lixisenatide once-daily add-on treatment to basal insulin therapy ±oral antidiabetic drugs (OADs) among type 2 diabetes (T2DM) patients subdivided into groups, based on their baseline body mass indices (BMI).

**Methods:**

Data of patients treated with lixisenatide were extracted from the modified intent-to-treat populations of the trials. Patients were subdivided into 4 groups based on baseline BMI category (BMIs <25, 25–<30, 30–<35, and ≥35 kg/m^2^). At the unadjusted data level, efficacy and safety endpoints were evaluated and compared among study cohorts. Additionally, multivariable regression analyses were used to specify key patient characteristics and then assess the adjusted outcomes.

**Results:**

Of the 662 T2DM patients, the mean changes in HbA1c (−0.63 to −0.73 %, p = 0.88) and FPG levels (−3.9 to 3.2 mg/dL, p = 0.60) were not significantly different among the different BMI groups. The proportions of T2DM patients that achieved HbA1c <7 % ranged between 34.7 and 46.8 %. After adjusted for patient characteristics, T2DM patients in the lowest BMI group relative to those in the highest BMI group had a smaller reduction in HbA1c during the trial periods (difference: 0.32 %, confidence interval: 0.10, 0.53, p = 0.005) and were less likely to achieve HbA1c <7 %.

**Conclusions:**

The findings of this analysis of the GetGoal clinical trials suggest that lixisenatide may be a good treatment option for optimizing glycemic control in patients unable to achieve their HbA1c target on basal insulin therapy ±OADs, regardless of BMI category.

## Background

Type 2 diabetes mellitus (T2DM) is a metabolic disease resulting from insulin resistance and β-cell dysfunction, both of which worsen over time. It is not rare that the progression of T2DM requires the addition of other treatments to diet, exercise, or oral antidiabetic drugs (OADs). The addition of basal insulin therapy can improve glycemic control [1, 2]. However, different from fasting hyperglycemia, postprandial hyperglycemia is not well controlled with basal insulin therapy and may be the limiting factor for achieving optimal glycemic control for many T2DM patients [3, 4]. Other detriments of basal insulin therapy include risks for hypoglycemia and weight gain [2].

Lixisenatide is a glucagon-like peptide-1 (GLP-1) receptor agonist, of which its antidiabetic actions are primarily attributed to the stimulation of glucose-dependent insulin secretion via direct action on β cells [2]. Lixisenatide also delays gastric emptying and promotes satiety, thereby stimulating weight loss [2]. In addition, lixisenatide suppresses glucagon secretion, which accompanied with the delay in gastric emptying and the increase in β-cell glucose sensitivity contributes to a marked reduction in postprandial glucose (PPG) level [2, 5–7]. These features of lixisenatide make it a practical option in conjunction with basal insulin therapy, which contributes primarily to lowering fasting plasma glucose (FPG).

The efficacy and safety of lixisenatide treatment for T2DM were evaluated in the GetGoal clinical trial program comprised of 11 trials [2]. GetGoal-Duo1, GetGoal-L, and GetGoal-L-Asia evaluated the efficacy and safety of lixisenatide add-on treatment to basal insulin therapy ±OADs among patients with T2DM [5–7]. In the GetGoal-Duo1 trial; add-on treatment to basal insulin plus metformin alone or in combination with other OADs, and in the GetGoal-L trial; add-on treatment to basal insulin ±metformin, they were associated with significant reductions in HbA1c (mean difference: −0.3 %; p < 0.0001 and −0.4 %; p = 0.0002), PPG (2 h after a standardized meal test, mean difference: −57.6 mg/dL, p < 0.0001 and −68.4 mg/dL, p < 0.0001), and body weight (mean difference: −0.9 kg, p = 0.0012 and −1.3 kg, p < 0.0001), respectively, relative to placebo add-on treatment [5, 6]. In the GetGoal-L-Asia trial; add-on treatment to basal insulin ±sulfonylurea, it was associated with significant reductions in HbA1c (mean difference: −0.88 %; p < 0.0001) and PPG (mean difference: −140.9 mg/dL, p < 0.0001) and a trend for reduction in body weight (mean change: −0.38 kg, p = 0.0857) relative to placebo add-on treatment [7].

A study of the relationship between body mass index (BMI) and disease risk using two national surveys, NHANES and SHIELD, observed that increased BMI is a predictor of increased prevalence of diabetes as already known [8]. In particular, this study reported that while >75 % of patients with diabetes are overweight or obese, between 10 and 25 % are not overweight [8]. Furthermore, obesity-related diseases, including T2DM, occur at lower BMIs among persons in Asian countries [9]. It is therefore important to evaluate the efficacy and safety of antidiabetic treatments in the context of BMI subcategories. Using data from the GetGoal-Duo1, -L, and -L-Asia trials, the objectives of this study were to evaluate and compare the impact of lixisenatide once-daily add-on treatment to basal insulin therapy ± OADs among T2DM patients placed in different BMI categories at the baseline; BMI <25 kg/m^2^, 25–<30 kg/m^2^, 30–<35 kg/m^2^ and ≥35 kg/m^2^.

## Methods

### Study population

All modified intent-to-treat patients from the GetGoal-Duo1 (NCT00975286), -L (NCT00715624), and -L-Asia (NCT00866658) trials with baseline and endpoint HbA1c measurements and baseline BMI measurements were included in the analysis. All three trials evaluated within the intent-to-treat populations the efficacy and safety of lixisenatide add-on treatment to basal insulin therapy ±OADs vs. placebo [5–7]. The primary efficacy outcome was the absolute change in HbA1c from baseline to week 24 [5–7]. In the trials lixisenatide treatment consisted of a once-daily injection in a two-step dose-increase regimen (10 μg for 1 week, 15 μg for 1 week, and then 20 μg if tolerated) [5–7]. All trials were approved by the institutional review boards or ethics committees and were conducted in accordance with the principles of the Declaration of Helsinki and Good Clinical Practice guidelines. Further details of the trials can be found in each individual study publication [5–7]. For this analysis patients from all three trials who were originally randomized to lixisenatide add-on treatment to basal insulin therapy ±OADs were subdivided into 4 groups based on their baseline BMI category (BMIs <25, 25–<30, 30–<35, and ≥35 kg/m^2^).

### Demographics and clinical characteristics

Demographics and key patient clinical characteristics, including baseline BMI, body weight, HbA1c, FPG, PPG (standardized 2-h meal test), and basal insulin dose and durations of T2DM, OAD use, and insulin use were determined based on clinical trial data.

### Efficacy and safety outcomes

Efficacy outcomes included mean changes in baseline to endpoint measurements in HbA1c, BMI, weight, FPG, and PPG. Additionally, the proportions of T2DM patients in the different BMI groups that achieved and did not achieve HbA1c <7 % and FPG <110 mg/dL were determined. The safety outcomes for evaluation included the proportions of T2DM patients in the different BMI groups that experienced severe and symptomatic hypoglycemia (requiring blood glucose levels <60 mg/dL) during trial periods. Other outcomes evaluated during the trial periods included the proportions of T2DM patients in the different BMI groups that achieved the following composite endpoints: HbA1c <7 % and no symptomatic hypoglycemia, HbA1c <7 % and no severe hypoglycemia, HbA1c <7 % and no weight gain, HbA1c <7 % and no symptomatic hypoglycemia and no weight gain, and HbA1c <7 % and no severe hypoglycemia and no weight gain.

### Statistical analyses

Descriptive statistics were used to measure and describe patient demographics and clinical characteristics as well as the efficacy and safety outcome measurements. The study groups, defined by baseline BMI category, were compared to one another with p-values calculated using a χ^2^ test or ANOVA test where appropriate. A *p* value of 0.05 was used to determine the level of statistical significance.

### Multivariable regression analyses

Multivariable regression analyses were used to assess the efficacy and safety outcomes, while adjusting key patient characteristics. Generalized linear models were used to examine whether there were differences in changes in HbA1c, FPG, PPG, and body weight among the different BMI study groups treated with lixisenatide add-on treatment. Estimated differences, 95 % confidence limits, and p-values were reported. Logistic regressions were used to assess the relative odds of symptomatic hypoglycemia and of achieving composite endpoints among the different BMI study groups treated with lixisenatide add-on treatment. Odds ratios, 95 % confidence limits, and p-values were reported. Covariates in all of the regression analyses included age, gender, and baseline FPG, PPG, HbA1c, and basal insulin dose/weight, and durations of diabetes, OAD use, and insulin use. All statistical analyses were carried out using SAS^®^ 9.3 (Cary, NC, USA).

## Results

### Characteristics of study population

Of the 662 T2DM patients treated with lixisenatide add-on treatment to basal insulin ±OADs included in this analysis, 133 (20 %), 222 (34 %), 166 (25 %), and 141 (21 %) had a baseline BMI <25, 25–<30, 30–<35, and ≥35 kg/m^2^ respectively. Baseline characteristics of study groups are summarized in Table 1. Mean ages of study groups were not significantly different (range 56.5–58.0 years, p = 0.58). Greater proportions of females vs. males were grouped in higher baseline BMI groups. The mean durations of OAD use (5.6–6.7 years, p = 0.29) and insulin use (1.7–2.3 years, p = 0.35) were similar among study groups. However, T2DM duration increased as BMI decreased (BMI <25 kg/m^2^: 13.1 years, 25–<30 kg/m^2^: 12.4 years, 30–<35 kg/m^2^: 11.3 years, ≥35 kg/m^2^: 10.4 years, p = 0.0043).

**Table 1:** Baseline characteristics of study groups

	BMI <25 kg/m^2^	BMI 25–<30 kg/m^2^	BMI 30–<35 kg/m^2^	BMI ≥35 kg/m^2^	P value
Total patient count, n (%)	133 (20 %)	222 (34 %)	166 (25 %)	141 (21 %)	
Age (years), mean (SD)	57.2 (10.4)	58.0 (9.5)	57.3 (9.7)	56.5 (9.8)	0.58*
Gender					0.0495**
Female, n (%)	62 (46.6)	117 (52.7)	88 (53.0)	89 (63.1)	
Male, n (%)	71 (53.4)	105 (47.3)	78 (47.0)	52 (36.9)	
Baseline weight (kg), mean (SD)	61.0 (9.0)	73.8 (9.9)	88.9 (11.8)	110.4 (18.6)	<0.0001*
Baseline BMI (kg/m^2^), mean (SD)	23.1 (1.5)	27.6 (1.5)	32.4 (1.4)	40.3 (5.0)	<0.0001*
Diabetes duration (years), mean (SD)	13.1 (7.7)	12.4 (6.8)	11.3 (7.1)	10.4 (6.8)	0.0043*
OAD history (years), mean (SD)	5.9 (4.8)	6.4 (6.2)	6.7 (5.4)	5.6 (4.4)	0.29*
Insulin history (years), mean (SD)	2.3 (3.9)	2.2 (3.2)	1.7 (2.5)	1.9 (3.2)	0.35*

*BMI* body mass index, *SD* standard deviation, OAD oral antidiabetic drug

* Statistical significance of differences was determined by ANOVA

** Statistical significance of differences was determined by X^2^ test

### Efficacy of lixisenatide add-on treatment to basal insulin therapy ±OADs

In Table 2, baseline, week 24, and mean changes from baseline to week 24 of efficacy measurements are summarized for each BMI group of patients from the 3 GetGoal trials. The mean changes in HbA1c (−0.63 to −0.73 %, p = 0.88) and FPG levels (−3.9 to 3.2 mg/dL, p = 0.60) were not significantly different among the different BMI groups. At the unadjusted state, the mean reductions in PPG level increased as BMI decreased (BMI <25 kg/m^2^: −126.6 mg/dL, 25–<30 kg/m^2^: −103.5 mg/dL, 30–<35 kg/m^2^: −82.0 mg/dL, ≥35 kg/m^2^: −67.2 mg/dL, p < 0.0001). Greater baseline basal insulin doses were observed as BMI increased, but the mean changes in basal insulin doses over trial periods did not significantly differ among BMI groups (p = 0.72). Mean changes in body weight (p = 0.06) and BMI (p = 0.07) did not reach the significance level among BMI groups.

**Table 2:** Clinical responses of study groups to lixisenatide add-on treatment to basal insulin therapy ± oral antidiabetic drugs

Subgroups	BMI <25 kg/m^2^	BMI 25–<30 kg/m^2^	BMI 30–<35 kg/m^2^	BMI ≥35 kg/m^2^	p value
HbA1c, % (SD)					
Baseline	8.23 (0.81)	8.24 (0.82)	8.06 (0.92)	8.06 (0.84)	0.07*
Week 24	7.61 (1.25)	7.56 (1.17)	7.38 (1.07)	7.33 (1.09)	0.10*
Mean change from baseline	−0.63 (1.19)	−0.68 (1.08)	−0.68 (0.92)	−0.73 (0.92)	0.88*
Patients with endpoint HbA1c n (%)					0.09**
<7 %	47 (35.3)	77 (34.7)	69 (41.6)	66 (46.8)	
≥7 %	86 (64.7)	145 (65.3)	97 (58.4)	75 (53.2)	
FPG mg/dL (SD)					
Baseline	129.2 (41.1)	133.7 (44.2)	136.5 (40.7)	137.7 (41.7)	0.35*
Week 24	132.5 (46.9)	135.4 (54.2)	132.7 (42.5)	135.6 (45.3)	0.90*
Mean change from baseline	3.2 (52.7)	1.7 (59.8)	−3.9 (46.6)	−2.1 (40.8)	0.60*
Patients with endpoint FPG n (%)					0.63**
<110 mg/dL	48 (36.6)	81 (37.2)	54 (32.5)	44 (31.7)	
≥110 mg/dL	83 (63.4)	137 (62.8)	112 (67.5)	95 (68.4)	
PPG mg/dL (SD)^a^					
Baseline	301.5 (75.2)	287.5 (77.3)	279.0 (82.2)	252.6 (75.2)	<0.0001*
Week 24	175.0 (86.8)	184.7 (83.8)	197.3 (82.0)	185.5 (68.9)	0.15*
Mean change from baseline	−126.6 (109.7)	−103.5 (109.2)	−82.0 (103.1)	−67.2 (84.0)	<0.0001*
Percent change from baseline	−42 %	−36 %	−29 %	−27 %	
Basal insulin dose U (SD)					
Baseline	26.2 (11.6)	36.5 (15.5)	50.3 (24.8)	63.9 (41.2)	<0.0001*
Week 24	25.5 (12.1)	36.2 (17.0)	50.4 (26.1)	62.1 (31.6)	<0.0001*
Mean change from baseline	−0.7 (4.8)	−0.3 (6.8)	0.2 (10.0)	−1.8 (29.8)	0.72*
Body weight kg (SD)					
Baseline	61.0 (9.0)	73.8 (9.9)	88.9 (11.8)	110.4 (18.6)	<0.0001*
Week 24	60.8 (9.3)	73.1 (10.0)	87.9 (12.2)	109.4 (19.1)	<0.0001*
Mean change from baseline	−0.2 (2.0)	−0.7 (2.4)	−1.0 (3.3)	−1.0 (3.5)	0.06*
BMI kg/m^2^ (SD)					
Baseline	23.1 (1.5)	27.6 (1.5)	32.4 (1.4)	40.3 (5.0)	
Week 24	23.1 (1.7)	27.4 (1.7)	32.0 (1.8)	39.9 (5.3)	<0.0001*
Mean change from baseline	−0.1 (0.8)	−0.2 (0.9)	−0.4 (1.2)	−0.4 (1.3)	0.07*

*BMI* body mass index, *FPG* fasting plasma glucose, *PPG* postprandial blood glucose

Data are mean (SD = standard deviation) unless otherwise stated

^a^2 h after a standardized meal test

* Statistical significance of differences was determined by ANOVA

** Statistical significance of differences was determined by χ^2^ test

**Fig. 1:**
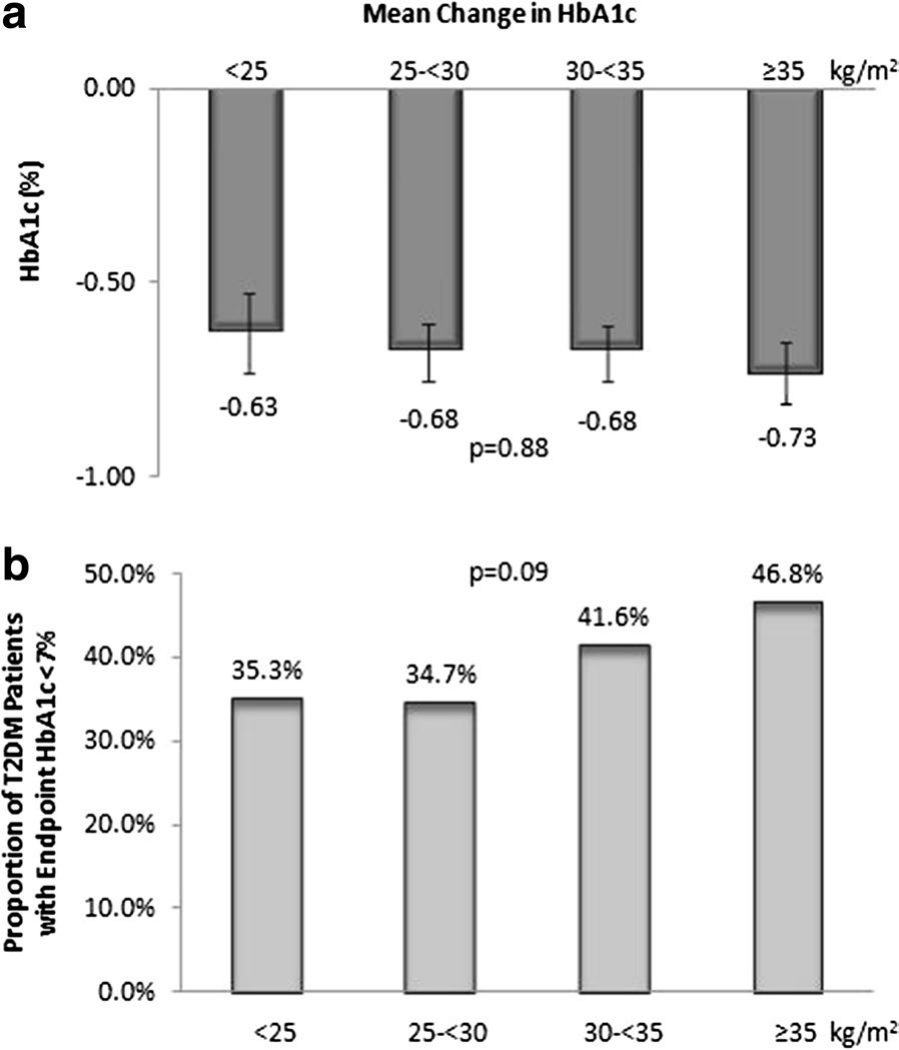
Mean Change in HbA1c of Study Groups (**a**) and Proportions of Study Groups that Achieved an Endpoint HbA1c <7 % (**b**). Statistical significance of differences in mean changes in HbA1c was determined by ANOVA with standard error presented and that of the differences in the proportions of T2DM patients with an endpoint HbA1c <7 % was determined by χ^2^ test

The proportions of T2DM patients that achieved an HbA1c <7 % ranged between 34.7 and 46.8 % and trended (p = 0.09) to be greater for the higher BMI groups, but did not reach the significance level (Fig. 1b). The proportions of T2DM patients in different BMI groups that achieved the evaluated composite endpoints also did not significantly differ, but trended to be greater for the higher BMI groups (Table 3).

**Table 3:** Frequency of achievement of composite endpoints among study groups

	BMI <25 kg/m^2^	BMI 25–<30 kg/m^2^	BMI 30–<35 kg/m^2^	BMI ≥35 kg/m^2^	p value
HbA1c <7 % and no symptomatic hypoglycemia, n (%)	26 (19.6)	58 (26.1)	49 (29.5)	42 (29.8)	0.18
HbA1c <7 % and no severe hypoglycemia, n (%)	47 (35.3)	76 (34.2)	69 (41.6)	65 (46.1)	0.10
HbA1c <7 % and no weight gain, n (%)	31 (23.3)	54 (24.3)	48 (28.9)	49 (34.8)	0.10
HbA1c <7 %, no symptomatic hypoglycemia, and no weight gain, n (%)	16 (12.0)	42 (18.9)	36 (21.7)	29 (20.6)	0.16
HbA1c <7 %, no severe hypoglycemia, and no weight gain, n, (%)	31 (23.3)	54 (24.3)	48 (28.9)	48 (34.0)	0.14

*BMI* body mass index; Statistical significance was determined by χ^2^ test

### Safety of lixisenatide add-on treatment to basal insulin therapy ±OADs

The frequency of severe hypoglycemia was low for all BMI groups and did not significantly differ among the BMI groups (Table 4). The frequency of symptomatic hypoglycemia (requiring blood glucose level <60 mg/dL) ranged between 24.1 and 32.3 % and also did not significantly differ among the BMI groups (Table 4).

**Table 4:** Frequency of hypoglycemia among study groups

	BMI <25 kg/m^2^	BMI 25–<30 kg/m^2^	BMI 30–<35 kg/m^2^	BMI ≥35 kg/m^2^	p value
Severe hypoglycemia, n (%)	1 (0.8)	2 (0.9)	0 (0.0)	2 (1.4)	0.54**
Events/patient-year mean (SD)	0.05 (0.6)	0.2 (3.5)	0.0 (0.0)	0.03 (0.3)	0.63*
Symptomatic hypoglycemia^a^, n (%)	43 (32.3)	62 (27.9)	43 (25.9)	34 (24.1)	0.46**
Events/patient-year mean (SD)	2.2 (4.4)	2.1 (6.3)	1.8 (4.9)	1.6 (6.7)	0.81*

*SD* standard deviation

^a^Requiring blood glucose <60 mg/dL

* Statistical significance of differences was determined by ANOVA

** Statistical significance of differences was determined by χ^2^ test

### Multivariable regression results

A summary of the significant multivariable regression results of T2DM patients treated with lixisenatide add-on treatment to basal insulin therapy ±OADs placed in different BMI groups is presented in Table 5. After adjusted for patient characteristics, T2DM patients in the lowest BMI group (<25 kg/m^2^) relative to the highest BMI group (≥35 kg/m^2^) had a smaller reduction in HbA1c during trial periods (difference: 0.32 %, CI 0.10, 0.53, p = 0.005). Changes in FPG, PPG, and body weight were not significantly different among the different BMI study groups at the adjusted level. Also, the likelihood of symptomatic hypoglycemia was similar among the different BMI groups treated with lixisenatide add-on treatment. T2DM patients in the lower BMI groups (<25 kg/m^2^, BMI 25–<30 kg/m^2^) relative to the highest BMI group (≥35 kg/m^2^) had lesser likelihoods of achieving HbA1c <7 % during trial periods (<25 kg/m^2^ OR 0.48, CI 0.27, 0.86, p = 0.014; BMI 25–<30 kg/m^2^ OR 0.59, CI 0.37, 0.92, p = 0.020). Additionally, T2DM patients in the lowest BMI group (<25 kg/m^2^) relative to the highest BMI group (≥35 kg/m^2^) had lesser likelihoods of achieving an HbA1c <7 % with no weight gain (OR 0.47, CI 0.26, 0.88, p = 0.018) and of achieving an HbA1c <7 % with no severe hypoglycemia and no weight gain (OR 0.47, CI 0.26, 0.88, p = 0.018) during trial periods.

**Table 5:** Summary of multivariable regression results of type 2 diabetes patients treated with lixisenatide add-on treatment to basal insulin therapy ± oral antidiabetic drugs placed in different body mass index (BMI) groups based on their baseline BMI

	Estimated difference	95 % Confidence limits		P-value
		Lower	Upper	
Change in HbA1c (%)				
BMI <25 kg/m^2^ vs. BMI ≥35 kg/m^2^	0.32	0.10	0.53	0.005
BMI 25–<30 kg/m^2^ vs. BMI ≥35 kg/m^2^	0.09	−0.09	0.26	0.32
BMI 30–<35 kg/m^2^ vs. BMI ≥35 kg/m^2^	−0.01	−0.18	0.17	0.94

	Odds ratio	Lower	Upper	p-value
Endpoint HbA1c <7 %				
BMI <25 kg/m^2^ vs. BMI ≥35 kg/m^2^	0.48	0.27	0.86	0.014
BMI 25–<30 kg/m^2^ vs. BMI ≥35 kg/m^2^	0.59	0.37	0.92	0.020
BMI 30–<35 kg/m^2^ vs. BMI ≥35 kg/m^2^	0.82	0.52	1.30	0.40
Endpoint HbA1c <7 % and no weight gain				
BMI <25 kg/m^2^ vs. BMI ≥35 kg/m^2^	0.47	0.26	0.88	0.018
BMI 25–<30 kg/m^2^ vs. BMI ≥35 kg/m^2^	0.72	0.45	1.15	0.17
BMI 30–<35 kg/m^2^ vs. BMI ≥35 kg/m^2^	0.79	0.49	1.29	0.35
HbA1c <7 %, no severe hypoglycemia, and no weight gain				
BMI <25 kg/m^2^ vs. BMI ≥35 kg/m^2^	0.47	0.26	0.88	0.018
BMI 25–<30 kg/m^2^ vs. BMI ≥35 kg/m^2^	0.72	0.45	1.15	0.17
BMI 30–<35 kg/m^2^ vs. BMI ≥35 kg/m^2^	0.79	0.49	1.29	0.35

## Discussion

The results of this analysis demonstrate that lixisenatide once-daily add-on treatment to basal insulin therapy ±OADs reduces HbA1c and PPG levels in T2DM patients across all BMI categories. The mean reductions in PPG levels were greater for T2DM patients categorized in lower BMI groups, but after adjusted for other patient characteristics they were not significantly different from the other BMI groups. In regard to HbA1c levels, after the adjustment the mean reduction was smaller for T2DM patients of normal weight relative to severely obese T2DM patients. Additionally, those with greater obesity were more likely to achieve an HbA1c <7 %. Greater obesity also correlated with a lesser duration of T2DM. Although, in the subgroup of patients with BMI <25 kg/m^2^ there was a greater proportion of Asian patients, which may have in part influenced the assessment of the relationship between T2DM duration and BMI, the trend of decreasing T2DM duration with increasing of BMI was observed across all BMI subgroups. The potentially greater efficacy of lixisenatide add-on treatment among those with severe obesity may in part be attributed to a less progressed state of diabetes. These observations may require further study for confirmation.

Lixisenatide treatment had a small effect on FPG level; however, this was expected as prior to initiating lixisenatide treatment, insulin therapy was stabilized and FPG levels were controlled among patients in the GetGoal trials [5–7]. Lixisenatide treatment was associated with a significant reduction in PPG levels, which led to better HbA1c control [5–7]. The relative contribution of postprandial hyperglycemia on overall glycemic control has been shown in other studies to contribute between 70 and 80 % to overall glycemic exposure in patients with relatively good glycemic control and to contribute less (40 %) in patients with poor glycemic control [10, 11]. Riddle et al. reported that targeting postprandial hyperglycemia is more critical in T2DM patients on basal insulin therapy with HbA1c not at target, as it is the main contributor to hyperglycemic exposure [12, 13]. The substantial lowering of PPG level associated with lixisenatide treatment may also potentially provide a cardiovascular benefit [14–16]. The International Diabetes Federation has recognized the importance of PPG control in reducing cardiovascular disease risks, in addition to other diabetic complications, and recommended lowering of postprandial glycemia as a major focus of T2DM management [17]. Further results of the influence of lixisenatide on cardiovascular outcomes are expected in 2015 after completion of the multicenter ELIXA study (Evaluation of Cardiovascular Outcomes in Patients with Type 2 Diabetes after Acute Coronary Syndrome During Treatment with AVE0010 [Lixisenatide], NCT01147250) [18].

A patient’s BMI did not significantly influence the frequency of hypoglycemia among lixisenatide treated patients. Hypoglycemia has been correlated with higher weight-based insulin doses in other studies, but this was not the case in this analysis [19]. In fact, among T2DM patients in higher BMI groups basal insulin doses were significantly greater; however the frequency of symptomatic hypoglycemia trended to decrease as BMI increased. Also, large changes in insulin doses did not occur among the different BMI study groups treated with lixisenatide add-on treatment during trial periods, which may be attributed in part to the design of these GetGoal trials, such that basal insulin doses were stabilized during the baseline periods prior to lixisenatide add-on treatment [5–7]. The stabilization of insulin doses during the baseline periods may have also contributed to the fact that a patient’s BMI did not significantly influence the frequency of hypoglycemia among lixisenatide treated patients.

Although not significant there was a trend observed in this analysis that having a greater baseline BMI is associated with a greater decrease in weight with lixisenatide add-on treatment. Thus, it is unlikely that normal weight T2DM patients are at risk for excessive weight loss when treated with lixisenatide add-on treatment. A smaller change in body weight was observed among patients in the subgroup with BMI <25 kg/m^2^ and may be related to that more of these patients were treated with sulfonylurea, which can be associated with weight gain [7]. Due to the limitation of the small number of clinical trials and small sample sizes, whether the use of sulfonylurea vs. other OADs or metformin added on to basal insulin may impact the weight change among lixisenatide users may require additional future studies.

### Limitations

There were trends for BMI to influence some efficacy measurements, which may be significant in studies with larger sample sizes (e.g. proportion of T2DM patients with HbA1c <7 % at trial endpoint at the unadjusted level). Inclusion of patients from the GetGoal-L-Asia trial in this analysis led to having an over representation of patients with lower BMIs, as the mean BMI of T2DM patients treated with lixisenatide in the GetGoal-Duo1, -L, and L-Asia trials were 32.0, 31.9, and 25.4 kg/m^2^, respectively. Additionally, patient data were extracted only from the lixisenatide treatment arms of the GetGoal trials. The results of this analysis may be further limited by the patient types and trial designs as evaluated in the GetGoal-Duo1, -L, and L-Asia trials. Furthermore, the impact of lixisenatide add-on treatment on blood pressure was not evaluated in this analysis since detailed data on blood pressure were not reported in the original GetGoal-Duo1, -L, and -L-Asia trials [5–7].

## Conclusions

The findings of these analyses of recent randomized clinical trials suggest that lixisenatide may be a good treatment option for optimizing glycemic control in T2DM patients unable to achieve their HbA1c glycemic target on basal insulin ±OADs, regardless of BMI.

## Abbreviations

BMI: body mass index
FPG: fasting plasma glucose
HbA1c: glycated hemoglobin
OADs: oral antidiabetic drugs
PPG: postprandial glucose
T2DM: type 2 diabetes
